# Contribution of Advanced Glycation End Products to PCOS Key Elements: A Narrative Review

**DOI:** 10.3390/nu14173578

**Published:** 2022-08-30

**Authors:** Marco Mouanness, Henry Nava, Christelle Dagher, Zaher Merhi

**Affiliations:** 1 Rejuvenating Fertility Center, New York, NY 10019, USA; 2 Department of Obstetrics and Gynecology, American University of Beirut Medical Center, Beirut P.O. Box 100, Lebanon; 3 Division of Reproductive Endocrinology and Infertility, Department of Obstetrics and Gynecology, SUNY Downstate Health Sciences University, Brooklyn, NY 11203, USA; 4 Division of Reproductive Endocrinology and Infertility, Department of Obstetrics and Gynecology, Maimonides Medical Center, Brooklyn, NY 11219, USA

**Keywords:** advanced glycation end products, PCOS, ovaries, insulin resistance, obesity, hyperandrogenism

## Abstract

In the last decade, data has suggested that dietary advanced glycation end products (AGEs) play an important role in both reproductive and metabolic dysfunctions associated with polycystic ovary syndrome (PCOS). AGEs are highly reactive molecules that are formed by the non-enzymatic glycation process between reducing sugars and proteins, lipids, or nucleic acids. They can be formed endogenously under normal metabolic conditions or under abnormal situations such as diabetes, renal disease, and other inflammatory disorders. Bodily AGEs can also accumulate from exogenous dietary sources particularly when ingested food is cooked and processed under high-temperature conditions, such as frying, baking, or grilling. Women with PCOS have elevated levels of serum AGEs that are associated with insulin resistance and obesity and that leads to a high deposition of AGEs in the ovarian tissue causing anovulation and hyperandrogenism. This review will describe new data relevant to the role of AGEs in several key elements of PCOS phenotype and pathophysiology. Those elements include ovarian dysfunction, hyperandrogenemia, insulin resistance, and obesity. The literature findings to date suggest that targeting AGEs and their cellular actions could represent a novel approach to treating PCOS symptoms.

## 1. Introduction

Polycystic ovary syndrome (PCOS), along with its reproductive and metabolic dysfunctions, is the one of the most common endocrine disorders affecting, according to some studies, 4 to 20% of reproductive-aged women [1]. The most common symptoms of PCOS include oligomenorrhea, hirsutism, insulin resistance (IR), obesity, and metabolic syndrome [2,3]. In the United States, the Rotterdam criteria are used for the diagnosis of PCOS where two out of the three of the following criteria need to be met: (1) oligo-anovulation; (2) hyperandrogenism (elevated levels of male hormones), or hirsutism, or acne; (3) polycystic ovaries looking on ultrasound (≥12 follicles measuring 2–9 mm in diameter and/or an ovarian volume >10 mL in at least one ovary) [4]. PCOS is a diagnosis of exclusion [5,6], and is made after ruling out other etiologies such as hypothyroidism, hyperprolactinemia, Cushing’s syndrome, non-classic adrenal hyperplasia, and others [5].

The etiology of PCOS is still unknown but has been linked to different hypotheses including environmental and genetic factors, which can cause an abnormal function in other metabolic pathways in the human body such as IR, fat tissue dysregulation, abnormal steroid hormones production, and hypothalamic-pituitary-ovarian malfunction [1].

In the last decade, several studies have shown that the reactive molecules, called advanced glycation end products (AGEs), contribute to the etiology of PCOS [7,8]. AGEs are created through non-ezymatic reactions between reducing sugars and free amino groups of proteins, lipids, or nucleic acids [9]. The endogenous formation of AGEs in the body takes several days to several weeks to complete with final AGEs’ concentration depending on the half-life of the glycated protein product; on the other hand there is physiologic clearance of those systemic AGEs [10]. Reactive carbonyl groups are constantly being produced via normal metabolism but when its production exceeds its clearance, AGEs start to accumulate in the blood and the tissues causing cellular oxidative stress and inflammation [10]. Their endogenous production increases progressively with aging, and there is a rapid acceleration of their production due to underlying medical conditions such as diabetes, cardiovascular disease, renal disease, Alzheimer’s, PCOS, and others [11,12,13,14,15]. In addition to the endogenous formation of AGEs, exogenous sources such as diet and smoking significantly increase body AGEs [16,17,18]. AGEs are well known to exist at high concentrations in foods that are processed under high-temperature conditions, such as frying, baking, and grilling [18]. Additionally, bodily exogenous AGEs originate from the consumption beverages rich in adducts, as well as the inhalation of combustion products, such as smoking and car exhaust [19].

AGEs are a family that include a variety of molecules, each with their own structure, characteristics, effect, and metabolic fate [20,21]. The characteristics and structure of each of the AGEs depend on whether they originate from an exogenous or an endogenous source, the type of precursor from which they originate, and whether they are in a free form or protein-bound form [20,21]. The structures of exogenous dietary AGEs are more complex and heterogeneous than to the endogenous AGEs [20,21]. However, once they enter the circulation and the body tissue, they become undistinguishable in terms of structure and function [20,21]. Pentosidine and N-carboxymethyl-lysine (CML) are well characterized, frequently studied, and used as markers of AGE accumulation in various tissues [8,22,23].

In the last decade, data has suggested that dietary AGEs play an important role in both the reproductive and metabolic dysfunctions associated with PCOS. This review will present new data relevant to the role of AGEs in several key elements of PCOS which include ovarian dysfunction, hyperandrogenemia, IR, and obesity.

## 2. Search Strategy and Data Extraction

A PubMed search was performed for all available basic science, animal studies, and clinical peer-reviewed articles including prospective, retrospective, randomized trials, and review articles published in English. The authors used the key words “advanced glycation end products and ovarian dysfunction and PCOS” which yielded 20 manuscripts, the key words “advanced glycation end products and hyperandrogenemia and PCOS” which yielded 4 manuscripts, the key words “advanced glycation end products and IR and PCOS” which yielded 26 manuscripts, and the key words “advanced glycation end products and obesity and PCOS” which yielded 13 manuscripts. The resulting manuscripts were reviewed by all co-authors and the data were extracted from the text, tables, and graphs within the manuscripts to complete this narrative review.

## 3. AGEs and Ovarian Dysfunction in PCOS

**Figure 1:**
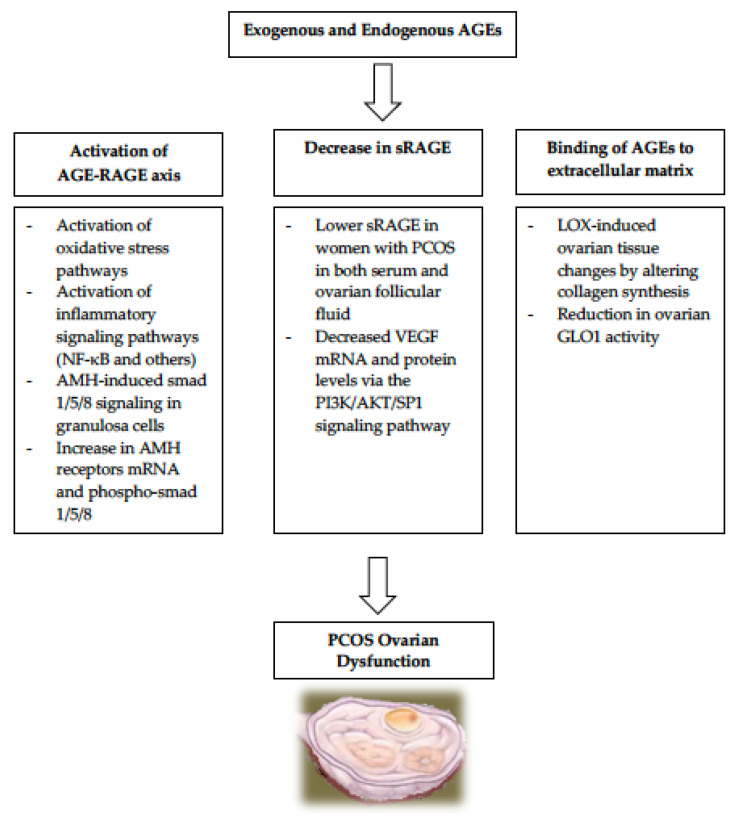
Systemic AGEs can be formed either endogenously or exogenously in women with PCOS. They contribute to the development of several key elements in PCOS via multiple pathways. NF-KB, nuclear factor kappa B; AMH, anti-mullerian hormone; mRNA, messenger RNA; PCOS, polycystic ovarian syndrome; LOX, lysyl oxidase; GLOX-1, glyoxalase 1.

Exogenous absorption of AGEs from heat processed diets exacerbates the already-elevated circulating AGEs levels found in PCOS patients [14,15,24,25,26,27]. Numerous studies in both humans and animals have proven that the elevated AGEs in PCOS, in both serum and ovaries and regardless of IR or obesity, contribute to the pathogenesis of the ovulatory cycles regardless of IR or obesity [14,15,24,25,26,27]. During normal folliculogenesis, multiple follicles undergo cellular proliferation and differentiation but most fail to complete their maturation process and become atretic, while only one dominant follicle attains full maturity then ovulates (DeGraafian Follicle). Unlike women without PCOS, women with PCOS have a large number of antral follicles which are mostly arrested at an early to mid-developmental state and unfrequently undergo maturation. The mechanistic pathways for AGEs’ contribution to the etiology of PCOS are summarized in Figure 1.

### 3.1. Activation of the AGE-RAGE Axis in PCOS

AGEs exert their actions via two main pathways, one is receptor dependent where AGEs bind to cell membrane receptors called RAGE (receptor for advanced glycation end products) [28], and the other is receptor independent. RAGE are known to be expressed in the reproductive system among other tissues [29,30,31]. The RAGE gene is located between the major histocompatibility complex 2 and 3 genes on chromosome 6 [32]. RAGE is a multiligand receptor that belongs to the immunoglobulin superfamily and it binds multiple families of ligands such as amyloid β peptide, S100/calgranulin protein, HMGB1, transthyretin, Mac-1, complement proteins (such as C3a and C1q), phosphadylserine, heparan sulfate, heat shock proteins, and others [33]. RAGE is a 35 kDa single transmembrane protein consisting of 394 amino acids in length, and it contains three domains: extracellular, transmembrane, and cytoplasmic [33]. The extracellular domain is large and composed of three distinct domains: immunoglobulin-like V (variable-type) domain and two constant domains named C1 and C2; the latter take part in the communication with the mediators of the transduction pathways [34]. The V and C1 domains form a structural and functional unit where most ligands bind [34]. Even though RAGE is the most-studied and best-characterized receptor, it is not the only one that binds AGE-modified proteins; other receptors include AGE Receptors 1, 2, and 3 (AGER-1, 2, 3) and macrophage scavenger receptor CD36 [35]. While AGER-2 participates in early AGE-activation, AGER 1 and 3 and CD36 receptors contribute to the detoxification pathways [36,37]. The biology of these AGE Receptors is not yet fully understood but consist of a different molecular structure with less harmful effects than RAGE [35].

The AGE–RAGE axis interaction activates several intracellular inflammatory signaling pathways [38,39], as well as oxidative stress-signaling pathways [28,40], leading to tissue damage also through a positive feedback loop that upregulates the expression of the RAGE receptor [36]. RAGE- and AGE-modified proteins have been shown to be differently expressed by immunohistochemistry in the ovaries of women with or without PCOS [24]. In the ovaries of women with PCOS, granulosa cells displayed a stronger RAGE expression compared to theca interna cells; however, the ovaries of women without PCOS exhibited equal staining between granulosa cells and theca interna cells [24]. Additionally, NF-κB p65 subunit (inflammatory signaling pathway) was only observed in the nuclei of granulosa cells of the ovaries of women with PCOS [24]. These findings suggest that these harmful molecules and the pro-inflammatory multi-ligand receptor RAGE have a pathological significance in reproductive abnormalities in ovarian dysfunction in PCOS.

An important hormone that plays a role in folliculogenesis is the anti-mullerian hormone (AMH) which plays an inhibitory role during folliculogenesis by suppressing the differentiation of granulosa cells, thus preventing follicles at earlier stages of development from maturation and atresia [41]. AMH acts mainly via intracellular phosphorylation of smad 1/5/8 [42,43]. It is well established that women with PCOS have abnormally elevated serum and ovarian levels of AMH which could lead to abnormal folliculogenesis and anovulation [44], in addition to elevated levels of AGEs [24]. Additionally, women with PCOS have abnormally elevated AMH-induced smad 1/5/8 signaling in granulosa cells and the exposure of human granulosa cells to AGEs in vitro has been shown to activate RAGE and lead to a significant increase in the AMH receptor mRNA and in the phospho-smad 1/5/8 [45]. This common PCOS-AGEs-AMH link highly suggests that AGEs could be one of the intermediary players responsible for the high-AMH production and for the AMHs heightened intracellular action, ultimately leading to PCOS-related anovulation.

### 3.2. Relationship of sRAGE to PCOS

In the receptor-independent actions, AGEs bind directly to the extracellular matrix of the tissues, thus altering their function [46,47]. There are two RAGE isoforms known as soluble RAGEs: the cleaved form, known as sRAGE which is generated by proteolytic cleavage of membrane-bound RAGE by the metalloproteases ADAM-10 and MMP-9; while the second form is called esRAGE (endogenous secretory RAGE) which results from alternative splicing of RAGE pre-mRNA [48]. The sRAGE receptors circulate in the blood and are present in bodily fluids (such as follicular fluid) and act as a neutralizer to AGEs’ effects [49], by preventing their binding to RAGE, and therefore dampening their pro-inflammatory action [50,51,52]. The sRAGE has been extensively studied in both the circulation and in body fluid (such as follicular fluid) because it can be easily quantified by ELISA [49,53,54]. Thus, it has been used as a biological marker of several pathologies such as diabetes, atherosclerosis, and others [55]. Studies have shown that women with PCOS had significantly lower sRAGE levels in their follicular fluid compared to women without PCOS [49,56,57]. One mechanism by which sRAGE has been shown to be protective to the ovaries of women with PCOS is via VEGF, which is known to be elevated in women with PCOS [49]. Culturing ovarian granulosa cells isolated from women with PCOS were treated in vitro with different concentrations of sRAGE, and the results showed that VEGF mRNA and protein levels decreased in a dose-dependent fashion in these cells compared with control ovarian granulosa cells (cells not exposed to sRAGE), this effect was most likely via the PI3K/AKT/SP1 signaling pathway [49,53].

### 3.3. AGEs Binding to Extracellular Matrix in PCOS

The accumulation of ovarian AGEs in women with PCOS causes dysregulation extracellular matrix organization within the ovarian follicles altering several enzymes and hormones involved in folliculogenesis:

(A) One enzyme important for normal folliculogenesis is LOX (lysyl oxidase), which is present in granulosa cells and in the para-follicular environment. It regulates the changes to the para-follicular connective tissue, helping the formation and maintenance of the extracellular matrix during follicular development [58,59]. LOX is overexpressed in the ovaries of women with PCOS, causing alteration in enzymes responsible for collagen synthesis and leading to increased volume and density of the ovarian stroma [60]. Since AGEs also induce LOX expression, it is very plausible that the elevated AGEs in women with PCOS are responsible for the LOX-induced ovarian tissue changes observed in PCOS [61].
(B) Another set of enzymes that play a role in protecting the ovarian function is the glyoxalase system. The two enzymes called glyoxalase 1 (GLO1) and glyoxalase 2 (GLO2), both of which are ubiquitously expressed to help in the protection against AGEs-related cellular damage by detoxifying one of the strong glycating agents called methylglyoxal [62]. Elevated levels of dietary AGEs have been linked to a reduction in ovarian GLO1 activity ultimately leading to ovarian dysfunction in PCOS [62]. These findings suggest that it is likely that AGEs play a role in the intraovarian pathophysiology in PCOS, partly via the glyoxalase system.

## 4. AGEs and Hyperandrogenism in PCOS

There is mounting evidence that points towards AGEs being a key player in the alteration of the biosynthesis of androgens in PCOS patients [15]. This effect is mediated via direct enzymatic change, but also via IR and induction of inflammatory changes. Regardless of BMI, serum AGEs’ levels are positively correlated with serum testosterone (T) levels in women with PCOS [15]. Additionally, women with PCOS given a high-AGE diet for two months had significantly higher serum AGEs, androstenedione, T, and free androgen index compared to women with PCOS on low-AGE diet for two months [63]. A human study evaluated the effect of AGE products on the function of human granulosa cells, and it found that the proliferation of human ovarian granulosa cells and the production of progesterone were inhibited by treatment with AGE products [64]. This occurred through downregulation of LH receptor/cAMP regulatory activity. The second part of that same study was conducted on rats where animals showed an irregular estrous cyclicity when they were subjected to AGE products and they had increased number of follicles/cysts, thin granulosa layer, and lower serum progesterone levels, all mimicking PCOS-like models. Human luteinized granulosa cells treated with AGEs in vitro had significant increase in CYP11A1, 3β-HSD, StAR, and CYP17A1 mRNA expression levels, but had no changes in CYP19A1 mRNA expression levels [65]. More studies in animal models showed that female rats given a high-AGE diet for six months had significantly elevated deposition of AGEs in their theca interna cells, increased RAGE expression in their granulosa cells, and higher blood T levels compared to rats on low-AGE diet group for six months [22]. Additionally, female rats on high-AGE diet for three months showed a significant elevation in serum T, but lower levels of estradiol and progesterone compared to rats on low-AGE diet for the same period [66]. There was also a positive correlation between serum AGEs and ovarian tissue weight, and between serum AGEs and serum T levels [22].

The opposite is also true, i.e., androgens themselves induce the action of AGEs by upregulating RAGE expression which is linked to endoplasmic reticulum (ER) stress [67]. A recent study showed that T increased RAGE expression and AGE accumulation in cultured human luteinized granulosa cells, and this was reduced by pretreatment with an agent that inhibits ER stress [67]. Furthermore, the elimination of the transcription factor C/EBP homologous protein (CHOP), an unfolded protein response factor activated by ER stress, inhibited T-induced RAGE expression and AGE accumulation. Additionally, the expression of RAGE and the accumulation of AGEs were upregulated in granulosa cells of women with PCOS and in DHEA-induced PCOS mouse model. The findings of this study suggest that hyperandrogenism in PCOS increases the expression of RAGE and accumulation of AGEs in the ovary through the activation of ER stress and that targeting the AGE–RAGE system, either by using a RAGE inhibitor or a clinically available ER stress inhibitor, may provide a novel therapeutic approach for PCOS. In summary, it seems that there is a positive reciprocal association between AGEs and hyperandrogenism; further solidifying the involvement of AGEs in the pathophysiology of PCOS.

## 5. AGEs and Insulin Resistance (IR) in PCOS

Insulin resistance has been independently linked to PCOS in a large portion of this patient population, considering about 75% of all cases, and this has been attributed to two factors: oxidative stress and inflammation [68,69,70]. The activation of intracellular pathways by the AGE–RAGE system leads to cellular oxidative stress via upregulation of markers of reactive oxygen species (ROS), activation of NADPH oxidase, and to cellular inflammatory state via the release of molecules such as tumor necrosis factor (TNF-α), interleukin-1 (IL-1), vascular adhesion molecule-1 (VCAM-1), interleukin-6 (IL-6), and others [40]. It is well known that IR and hyperinsulinemia play a role in the endocrine and reproductive disturbances observed in PCOS. Androgen excess itself can impair proper insulin actions, directly or by multiple changes at various sites in the body, especially in muscle and fat tissue [69]. Diets rich in fiber, complex carbohydrates, and unsaturated fat like the Mediterranean diet have been shown to have an anti-inflammatory effect, which causes decreased IR [70]. In a crossover trial, women were given a low- or high-AGE diet for two weeks, after which a hyperinsulinemic-euglycemic clamp and an intravenous glucose tolerance test were performed; and the results showed that high-AGE dietary intake caused a decrease in insulin sensitivity even though it did not cause changes in body weight or insulin secretion [71]. These results suggest that high dietary AGEs contribute to IR directly, regardless of adiposity status.

## 6. AGEs and Obesity in PCOS

Although there is no definite causality between AGEs and obesity, several studies have shown a positive correlation between the two [72]. Similar to IR, inflammation seems to be the main link between AGEs and obesity where it was demonstrated that the chronic exposure to a high dietary AGEs promotes chronic inflammation [69], ultimately leading to metabolic syndrome and obesity [72,73]. It has been reported that serum AGEs were significantly higher in obese humans suggesting a correlation with high-AGE diets [74]. An animal study where mice were given an isocaloric diet, with or without AGEs, demonstrated that mice on a diet that contained AGEs manifested increased adiposity and IR in their white adipose tissue, skeletal muscle, and liver. The glucose update was significantly lower in those tissues and was associated with significant changes in the insulin receptor, insulin receptor substrate (IRS)-1, and IRS-2 [75]. In a human study, overweight women on a low-AGE diet for four weeks had significantly lower-fasting serum insulin levels and lower HOMA-IR compared to overweight women on high-AGE diets [76]. A meta-analysis of 17 randomized controlled trials, where participants were blinded to the type of diet, examined the effects of low-AGE diets on cardiometabolic parameters and showed that although there were no changes in body weight, fasting glucose, 2-h glucose and insulin, or hemoglobin A1c, low-AGE diet lowered IR, total cholesterol, and low-density lipoprotein and caused a reduction in TNF-α, VCAM-1, 8-isoprostane, leptin, circulating AGEs, and endogenous receptor for AGEs in peripheral mononuclear cells [77].

Table 1 summarized the studies showing the effect of AGEs on insulin resistance, obesity, and hyperandrogenism in PCOS.

## 7. Pharmacological and Nutritional Therapeutic Options

In order to reduce oxidative stress, patients are usually counseled by their physicians to take supplements with antioxidant activities such as vitamins C, vitamin E, selenium, and others but little attention has been given to antiglycation measures in clinical practice [78], such as RAGE antagonists which could reduce oxidative stress by directly inhibiting the RAGE receptor activity [79]. Reducing the intake of AGE-containing diets has the most beneficial effects on metabolic and hormonal profiles as well as ovarian function in women with PCOS [80]. Furthermore, different diets have been proven to generate varying levels of AGEs. Low caloric Mediterranean diet [81], has been shown to reduce levels of carboxymethyl-lysine (CML) in obese premenopausal women. In line with this, the Mediterranean diet, rich in monounsaturated fatty acids and minimally processed food, was found to reduce postprandial oxidative stress and inflammation by modulating redox-state parameters, reducing AGEs levels and increasing AGER1 and Glox-1 mRNA levels compared with the high-saturated-fat diet [82]. Surprisingly, some studies have demonstrated an increased level of AGEs in omnivores/vegetarian diets compared to carnivorous diets but were not able to justify the mechanism behind it as vegetarian diets tend to require less cooking time and lower temperatures for cooking [83]. However, since eating a low-AGE diet could be challenging, there is a need for pharmacological options.

7.1. Metformin, a very commonly used anti-diabetic medication, has been shown to reduce the serum levels of pro-inflammatory markers and cause a subsequent decrease in systemic AGE following the intake over six-month period [16,27].
7.2. Women with PCOS and vitamin D deficiency who received vitamin D supplementation showed a significant decrease in the abnormally elevated serum AMH in PCOS and had an increased serum sRAGE levels suggesting that vitamin D supplementation in deficient women with PCOS could reduce the harmful effects of AGEs in PCOS [18,27].
7.3. Orlistat, a lipase inhibitor, has also been linked with decreased post-meal AGEs levels in women with PCOS as well as fasting insulin and testosterone concentrations [16].
7.4. Testosterone, which could be elevated in women with PCOS, increases RAGE expression and AGE accumulation in cultured human granulosa lutein cells (GLCs), and this was reduced by treatment with tauroursodeoxycholic acid (TUDCA), which acts as a chemical chaperone that dampens protein misfolding and improves ER stress. [67].
7.5. Recent studies have shown that L-Carnitine intake in women with PCOS showed improvements in hormonal and metabolic values, increased energy expenditure, and reduced lipids and body weight [80].

The inhibitors of AGEs’ formation (such as aminoguanide) and the inhibitors of AGEs’ absorption (such as AST-120) are compelling pathways for the reduction of AGEs and their inflammatory actions. A lot of other agents that act on reducing AGEs or their actions have been studied; some of those include pyridoxamine, benfotiamine, angiotensin-converting enzyme inhibitors, angiotensin receptor blockers, statins, ALT-711 (alagebrium) and, thiazolidinediones.

## 8. Conclusions

The role of AGEs in PCOS has gained great attention from researchers who are involved in female reproduction. The simple Maillard reaction, commonly known as protein glycation, occurs within the body under normal conditions as well as within foods prepared at high temperatures, leading to elevated serum levels of AGEs which are risk factors for various disorders including PCOS. The interaction of AGEs with their membrane receptors RAGE activates signaling pathways leading to an increase in oxidative stress, inflammation, ovulatory dysfunction, hyperandrogenism, IR, and obesity. In particular, AGE products exert a toxic effect on ovarian granulosa cells, especially on cell proliferation and hormone release. Several drugs such as metformin, aminoguanidine, benfotiamine, and others have been under investigation for preventing AGE-related dysfunctions [84,85]. Natural dietary agents with anti-AGE activity include medicinal plants such as green tea polyphenol compounds [86]. There is a need for more investigations pertaining to the incorporation of natural and synthetic anti-AGE dietary components in the treatment of PCOS symptoms. Finally, studying AGEs will help us in better understanding the physiology of PCOS and its phenotypes described above since most of us consume foods prepared at high temperatures (not easy to reduce their intake) which creates a flood of AGEs in vivo. Anti-AGEs drugs should be seriously considered as attractive options in the near future.

## Figures and Tables

**Table 1:** Summary of relevant studies pertaining to the role of AGEs in key elements of PCOS.

Authors and Study Type	Mode of Delivery of AGEs	Outcome	Comorbid Conditions
Po-Han Lin, et al.: Preclinical and Clinical [64]	- Five indented BSA-derived AGE products were used to evaluate their effect on the function of human granulosa cells. - The same study was conducted on rats where animals showed irregular estrous cyclicity.	- AGEs exert a toxic effect on ovarian granulosa cells, ovarian morphology, and the estrous cycle. - Rats had increased number of follicles/cysts, thin granulosa layer, and lower serum progesterone levels.	Mimic DHEA-induced PCOS phenotypes.
Diamanti-Kandarakis E, et al.: Preclinical [22]	Female rats given a high-AGE diet for six months.	Rats had significantly elevated deposition of AGEs in their theca interna cells, increased RAGE expression in their granulosa cells, and higher blood T levels compared to rats on low-AGE diet.	A positive correlation between serum AGEs and ovarian tissue weight, and between serum AGEs and serum T levels.
Azhary JMK, et al.: Clinical [67]	T increased RAGE expression and AGE accumulation in cultured human luteinized granulosa cells.	Androgens induced the action of AGEs by upregulating RAGE expression.	Reduced by pretreatment with an agent that inhibits ER stress.
De Courten B, et al.: Clinical [71]	Women were given a low- or a high-AGE diet for two weeks.	Decrease in insulin sensitivity.	No changes in body weight or insulin secretion.
Cai W, et al.: Preclinical [75]	Mice were given an isocaloric diet, with or without AGEs.	Mice on a diet that contains AGEs manifested increased adiposity and IR in their white adipose tissue, skeletal muscle, and liver.	Significant changes in insulin receptor.
Mark AB, et al.: Clinical [76]	Overweight women on a low-AGE diet for four weeks.	Had significantly lower fasting serum insulin levels and lower HOMA-IR compared to overweight women on high-AGE diet.	

Abbreviations: PCOS: polycystic ovary syndrome, DHEA: dehydroepiandrostenedione, BSA: bovine serum albumin, AGE: advanced glycation end product, IR: insulin resistance, T: testosterone, RAGE: receptor for advanced glycation end product, HOMA: homeostatic model assessment.

## Data Availability

Not applicable.
